# Supplementary material for: The genome sequence of the Vestal Cuckoo Bee, Bombus vestalis (Geoffroy, 1785)
Source: Wellcome Open Res. 2025 Apr 25;10:218. [Version 1] doi: 10.12688/wellcomeopenres.24004.1 (PMC12102655; doi:10.12688/wellcomeopenres.24004.1)
Supplement: Peer review history

# Reviewer report: Reviewer response for version 1

**DOI:** 10.21956/wellcomeopenres.26482.r122720

**Published:** 2025-05-23

**Authors:** Wiebke Sickel

**Affiliations:** 1 Thünen-Institute of Biodiversity, Braunschweig, Braunschweig, Germany

The data note presents the first genome assembly of the vestal cuckoo bee. The assembly is of high-quality, completeness and the methods are described in detail.

 I have two very small comments:

 (1) Under "Assembly curation", the author writes "The assembly was decontaminated using the Assembly Screenfor Cobionts and Contaminants (ASCC) pipeline (article in preparation)." - I wonder whether a repository or similar could be referenced at this stage?

 (2) Under "Wellcome Sanger Institute – Legal and Governance", the author writes "[...] the Darwin Tree of Life Partner agrees they will meet the legal and ethical requirements nd [sic] standards set out within this document in respect of all samples acquired for [...] - there is a typo, should read "and"

 Otherwise I recommend to index this data note.

Are sufficient details of methods and materials provided to allow replication by others?

Yes

Is the rationale for creating the dataset(s) clearly described?

Yes

Are the datasets clearly presented in a useable and accessible format?

Yes

Are the protocols appropriate and is the work technically sound?

Yes

Reviewer Expertise:

biodiversity, monitoring, molecular ecology

I confirm that I have read this submission and believe that I have an appropriate level of expertise to confirm that it is of an acceptable scientific standard.

# Reviewer report: Reviewer response for version 1

**DOI:** 10.21956/wellcomeopenres.26482.r122716

**Published:** 2025-05-13

**Authors:** Fatih Dikmen

**Affiliations:** 1 Istanbul University, Istanbul, Turkey

This data note presents a high-quality chromosome-level genome assembly for the Vestal Cuckoo Bee ( *Bombus vestalis*), which is part of the broader Darwin Tree of Life project. The authors describe the collection, sequencing, assembly, and annotation processes in comprehensive detail. The resulting genome spans 280.01 megabases with 91.96% of the assembly scaffolded into 25 chromosomal pseudomolecules, and includes a mitochondrial genome of 24.37 kilobases. Gene annotation identified 11,600 protein-coding genes. The paper successfully meets the standards of a data note, providing all necessary technical information without unnecessary speculation or interpretation.I recommend this data note for publication without revisions. The authors have produced a high-quality genome resource that will be valuable for future comparative genomic studies of bees, particularly social parasites. The assembly meets all technical quality standards, and the accompanying documentation is excellent. This resource will contribute significantly to  understanding the evolution and biology of social parasitism in bees.  

https://biorisk.pensoft.net/article/4749

Are sufficient details of methods and materials provided to allow replication by others?

Yes

Is the rationale for creating the dataset(s) clearly described?

Yes

Are the datasets clearly presented in a useable and accessible format?

Yes

Are the protocols appropriate and is the work technically sound?

Yes

Reviewer Expertise:

Entomology and Bee Taxonomy

I confirm that I have read this submission and believe that I have an appropriate level of expertise to confirm that it is of an acceptable scientific standard.
